# Healthcare-Associated Infection Prevention Interventions for Neonates in Resource-Limited Settings

**DOI:** 10.3389/fped.2022.919403

**Published:** 2022-07-07

**Authors:** Angela Dramowski, Marina Aucamp, Emily Beales, Adrie Bekker, Mark Frederic Cotton, Felicity C. Fitzgerald, Appiah-Korang Labi, Neal Russell, Jonathan Strysko, Andrew Whitelaw, Susan Coffin

**Affiliations:** ^1^Department of Paediatrics and Child Health, Faculty of Medicine and Health Sciences, Stellenbosch University, Cape Town, South Africa; ^2^Infection Prevention and Control Service, Mowbray Maternity Hospital, Cape Town, South Africa; ^3^Center for Neonatal and Pediatric Infection, St George's University of London, London, United Kingdom; ^4^Infection, Immunity and Inflammation, UCL Great Ormond Street Institute of Child Health, University College London, London, United Kingdom; ^5^Department of Medical Microbiology, University of Ghana Medical School, Accra, Ghana; ^6^Department of Paediatric and Adolescent Health, Princess Marina Hospital, Gaborone, Botswana; ^7^Department of Global Medicine, Children's Hospital of Philadelphia, Philadelphia, PA, United States; ^8^Division of Medical Microbiology, Faculty of Medicine and Health Sciences, Stellenbosch University, Stellenbosch, South Africa; ^9^National Health Laboratory Service, Tygerberg Hospital, Cape Town, South Africa; ^10^Division of Infectious Diseases, Department of Pediatrics, Children's Hospital of Philadelphia, Philadelphia, PA, United States

**Keywords:** neonate, healthcare-associated infection, antimicrobial resistance, resource-limited, care bundles

## Abstract

Healthcare-associated infections (HAIs) and antimicrobial-resistant (AMR) infections are leading causes of neonatal morbidity and mortality, contributing to an extended hospital stay and increased healthcare costs. Although the burden and impact of HAI/AMR in resource-limited neonatal units are substantial, there are few HAI/AMR prevention studies in these settings. We reviewed the mechanism of action and evidence supporting HAI/AMR prevention interventions, including care bundles, for hospitalized neonates in low- and middle-income countries (LMIC).

## Introduction

### Epidemiology of Neonatal Deaths in Low- and Middle-Income Countries

The neonatal period, defined as the first 28 days from birth, is the most vulnerable period in the life of a child, accounting for 47% of deaths in children under 5 years of age (1, 2). For neonates in LMIC, the risk of death is up to 11-fold greater than in high-income countries (2). Reducing neonatal mortality to below 12 per 1,000 live births globally by 2030 is a key target of the sustainable development goals (SDGs) (2). Despite substantial progress in reducing the annual number of global neonatal deaths from 5 to 2.5 million between 1990 and 2019, there has been no reduction in neonatal mortality in sub-Saharan Africa. Over 95% of the 2.5 million neonatal deaths each year now occur in LMICs, with the sub-Saharan African and South-East Asian regions contributing approximately 1 million deaths each, with 27 and 23 deaths per 1,000 live births, respectively (2).

### Contribution of Severe Bacterial Infection to Neonatal Mortality

Approximately, one-third of neonatal deaths annually (680,000) are caused by infections (3), notably severe bacterial infections (bloodstream infection, pneumonia, and meningitis). In LMIC, the proportion of neonatal infections that are healthcare-associated infections (HAIs) to total neonatal infections is uncertain, as few neonatal units in resource-limited settings conduct systematic surveillance (4). There is also evidence that many deaths in preterm neonates are attributable to sepsis, but are mislabeled as prematurity-related deaths due to a lack of access to accurate diagnostic tests (5). The burden and impact of neonatal HAI are substantial and are likely to increase due to growth in population, in-hospital births, and preterm delivery rates in sub-Saharan Africa and South-East Asia (6).

### Factors Contributing to Bacterial Colonization in LMIC Neonatal Units

In resource-limited settings, early and heavy neonatal bacterial pathogen colonization occurs through exposure to suboptimal water, sanitation, hygiene, infection prevention and control (IPC) practices (4, 7), and high rates of maternal colonization with multidrug-resistant organisms (MDROs) (8) There are numerous challenges to the implementation of IPC best practices in LMIC obstetric and neonatal units. These include overcrowding, shared equipment with inadequate reprocessing, inadequate environmental cleaning, suboptimal hand hygiene compliance, limited laboratory and capacity for HAI surveillance with delayed recognition of outbreaks, and high rates of antibiotic prescribing (4, 7, 9, 10). In such settings, sustained bacterial transmission pressure leads to MDRO colonization, with many neonates subsequently developing HAI after bacterial translocation, invasion, and dissemination (11).

We reviewed the literature to identify, categorize, and prioritize HAI prevention interventions for hospitalized neonates in LMIC, including care bundles and multimodal infection prevention programs. Below, we present our findings, categorized into four main domains of interventions: (a) promotion of colonization with normal flora, (b) prevention of colonization with pathogens, (c) maintenance of skin integrity, and (d) HAI surveillance, education, and advocacy (Table 1).

**Table 1:** Selected healthcare-associated infection (HAI) prevention bundles and programs implemented in low- and middle-income country (LMIC) neonatal units.

**Author, country, year**	**Study design, population**	**Bundle/program elements**	**Key findings**
Urzúa (141), Chile, 2017	Non-controlled before after study, all neonates, single neonatal ICU	Adjusted empiric and targeted antibiotic therapy, prompt discontinuation of antibiotics when sepsis was excluded, antibiotic restriction policies, “bundles” for invasive procedures	Incidence of LOS decreased from 14.3 to 8.5 per 1,000 live births (*p* < 0.01). Mortality rates and length of hospital stay were similar in both study periods
Mwananyanda (98), Zambia, 2019	Prospective cohort, single neonatal unit	IPC bundle including staff training, text message reminders, alcohol-based hand rub, enhanced environmental cleaning, and weekly bathing of babies >1,500 g with 2% chlorhexidine gluconate	Hospital-associated mortality was lower during the intervention than baseline period (18.0 vs. 23.6%). The incidence density rate of clinically suspected sepsis and the rate of HA-BSI with pathogen was also lower in the intervention than baseline period (both *p* < 0.01)
Dramowski (70), South Africa, 2021	Prospective, quasi-experimental study, single neonatal unit	Multimodal cleaning intervention for surfaces and equipment in a neonatal ward, including cleaning audits with feedback, cleaning checklists, in-room cleaning wipes and training of staff and mothers in cleaning methods	The proportion of surfaces and equipment exhibiting no bacterial growth increased between study phases (*p* = 0.007). The proportion of surfaces and equipment meeting the adenosine triphosphate (ATP) “cleanliness” threshold (<200 relative light units) increased over time (*p* = 0.002), as did the ultraviolet (UV) marker removal rate (*p* < 0.001). The neonatal bloodstream infection rate declined following implementation of the NeoCLEAN intervention, from 6.7 to 3.9/1,000 patient days between the baseline and intervention periods (*p* = 0.166)
Johnson (142), India, 2022	Prospective, quasi-experimental study, 4 neonatal units	Comprehensive Unit-based Safety Program (CUSP) including interventions for hand hygiene, aseptic technique for invasive procedures, medication administration and intravenous fluid preparation and administration	Overall, there was no statistically significant change in the monthly HA-BSI or mortality rates from baseline to the post-intervention period [RR 0.97 (95% CI 0.92–1.03)]. Hand hygiene compliance odds increased 6% per month, and staff completed insertion checklists for 68% of neonates with a central line. However improved patient safety culture domains were observed for: management support for patient safety; teamwork and organizational learning-continuous improvement.

*IPC, infection prevention and control; ICU, intensive care unit; LOS, late-onset sepsis; HA-BSI, healthcare-associated bloodstream infection*.

#### Promoting Colonization With Normal Flora

##### Kangaroo Mother Care and Breastmilk

Neonates are extremely vulnerable to HAIs owing to their immune system immaturity and poorly developed skin and gastrointestinal barriers. Both kangaroo mother care (KMC) and breast feeding are evidence-based interventions that significantly reduce HAI risk and length of hospital stay, promote growth, and enhance neonatal survival (12–15). Several meta-analyses concluded that KMC reduces in-hospital neonatal mortality by up to 40% in babies with birth weight <2,000 g, and is highly effective in reducing severe morbidity, particularly from infection (12, 13). A recent randomized trial provides new evidence that a further 25% reduction in preterm neonatal mortality can be achieved by initiating KMC immediately after birth, instead of after clinical stability is attained. Despite its major benefits, KMC is not widely adopted in many LMICs. Inadequate facilities and/or space, limited hospital budgets, inadequate staffing, lack of KMC policies, and the reluctance of families to accept extended hospitalization are the main barriers to KMC uptake in Africa (16–18).

Mother's breastmilk is critical to seeding the neonatal gut microbiota, providing passive immunity and accelerating mucosal immune development and maturation of the intestinal epithelium, contributing to intestinal barrier integrity and decreasing infection risk. Bioactive substances in breastmilk, such as immunoglobulins, immune cells, microbes, mucins, cytokines, and human milk oligosaccharides, promote the development of the neonate's immune system and reduce infection risk (15). Both KMC and exclusive breastmilk feeding are low-cost, feasible, and evidence-based interventions, but require the continuous, onsite presence of mothers at the hospital. To enable the full benefits of these interventions to be realized, neonatal units in LMIC must include “in-unit” beds and KMC facilities.

##### Probiotics

Despite extremely promising, neonatal probiotic interventions are not yet well-established options for IPC care bundles. Probiotics, such as *Bifidobacterium* spp. and *Lactobacillus* spp., are live microorganisms, which support neonatal gut microbiota colonization (19). Preterm neonates with immature gut development may benefit from early probiotic exposure to improve intestinal barrier integrity and modulate immune system development (20). A systematic review of 23 randomized controlled trials in preterm neonates in LMICs compared probiotics to placebo and found that probiotics-supplemented neonates experienced a reduced risk of necrotizing enterocolitis (NEC), late-onset sepsis, and all-cause mortality (21). Access to probiotics remains limited in LMIC neonatal units and more research is needed to identify the optimal combination of strains for HAI prevention. A similar product of interest is prebiotics, which can be produced and administered exogenously, resembling prebiotics that occurs naturally in breast milk. Prebiotics can promote colonization by commensal organisms, inhibit pathogen binding, and strengthen the intestinal barrier. Prebiotics and symbiotics (a combination of prebiotics and probiotics) have very little efficacy data in the neonatal population, and further trials are required (22).

##### Antibiotic Stewardship and Diagnostic Microbiology Laboratory Support to Neonatal Units

Neonates are at high risk of bacterial infection with high empiric antibiotic prescription rates in LMICs attributable to diagnostic uncertainty, low blood culture yields, and concerns for infection-related deaths. The adverse long-term effects of neonatal antibiotic exposure are increasingly recognized, including shifts in gastrointestinal microbiome composition and selection for resistance genes (23). This phenomenon occurs both in individual patients and in the neonatal unit environment resulting in multiple foci of antibiotic-resistant bacteria (24–26). For this reason, antimicrobial stewardship programs (ASP) are of particular importance in neonatal units, although data on program implementation in LMIC neonatal units are very limited (27–29).

Up to 16 times more neonates receive antibiotics for culture-negative sepsis than for blood culture-confirmed infection, with suspected early onset sepsis contributing the largest proportion (30). Prompt cessation of empiric antibiotics is therefore a priority for antimicrobial stewardship in LMIC neonatal units. This could be facilitated by aseptically collected blood cultures, adequate blood culture bottle inoculum volume (minimum 1 ml), and prompt antibiotic discontinuation in the absence of clear clinical, biochemical, or microbiological indicators of infection (30). A barrier to this is the time-to-detection of bacterial growth. Current estimates of 48 h are based on automated blood culture systems, which show better performance in yield and speed, but pose financial and logistic challenges compared to manual blood culture systems, which are largely used in LMICs (31). The time-to-detection of growth is not clearly defined for manual blood cultures, and further research and innovation are needed in manual blood culture methods (32). For culture-positive cases, antibiotic treatment should be tailored to the pathogen's susceptibility using the narrowest spectrum antibiotic available. Antibiotic stewardship committees to advise on difficult cases and provide antibiotic authorization may also curb unnecessary antibiotic use.

An additional and growing challenge in LMIC neonatal units is antimicrobial resistance (AMR), which contributes to an estimated 140,000 neonatal deaths annually (33). Inappropriate and unnecessary use of antimicrobials in neonatal units exacerbates the development of AMR and further limits options for the treatment of AMR infections. Acquisition of colonization with AMR pathogens and subsequent development of HAI is a particular problem in neonatal intensive care units (NICU), owing to higher use of invasive devices and higher rates of exposure to antibiotics. Strategies to slow the development of AMR in neonatal units must include both considerate antibiotic use (through ASP) and the adoption of strict infection prevention practices. However, the evidence base for effective ASP in LMIC neonatal units remains limited (27).

The infrastructure, services, and expertise of diagnostic microbiology laboratories are critical contributors to IPC and ASP (10). LMIC neonatal units, in particular, benefit from access to on-site or closely situated microbiology laboratories, owing to their high burden of HAI and frequent outbreaks (34). Additional important contributions of clinical microbiology services include rapid pathogen detection, identification of resistance patterns, development of antibiograms, outbreak investigation, diagnostic stewardship, and HAI surveillance (10).

#### Prevention of Colonization With Pathogens

##### Prevention of Vertical Pathogen Transmission in Labor

Many interventions to reduce vertical bacterial transmission and neonatal sepsis do not specifically target MDROs and reduce the transmission of antibiotic-resistant and susceptible pathogens. Two such interventions are prepartum chlorhexidine gluconate (CHG) vaginal cleaning and neonatal whole-body CHG bathing. To date, the results have been mixed, with one South African study of intrapartum 0.5% CHG vaginal wipes showing no change in early onset neonatal sepsis or transmission of Group B *Streptococcus* (35), while another study in Zimbabwe using 1% CHG wipes showed a 50% reduction in vaginal pathogen colonization compared to standard care (36). An ongoing clinical trial in Malawi (NeoVT-AMR), comparing CHG and octenidine, should provide further insights into the impact of vaginal wipes on pathogen transmission (37).

The World Health Organization (WHO) promotes the use of clean birth kits in LMICs, containing soap, razor blade, cord ties, alcohol swabs, plastic sheets, and gauze. A recent review showed that birth kits were associated with reductions in puerperal sepsis, neonatal tetanus, perinatal, neonatal, and infant mortality (38). Barriers to the widespread use of clean birth kits and antiseptics include the lack of awareness, cost, and availability.

Mode of delivery and intrapartum antibiotic prophylaxis (IAP) influence neonatal MDRO acquisition. Metagenomic profiling showed that neonates born by Cesarean section, as compared to vaginal birth, had greater gut colonization with potential pathogens (including *Klebsiella* species, *Enterobacter* species, and *Enterococcus* species). The authors speculated that pathogen colonization was driven by extended exposure to the hospital environment (i.e., prolonged in-hospital stay following Cesarean section) (39). In this and other studies, babies delivered vaginally who received IAP, also showed microbiota perturbation. Further research is needed to identify ways to modulate the impact of mode of delivery and IAP on neonatal bacterial colonization (40).

##### Decolonization Interventions

Mupirocin for methicillin-resistant *Staphylococcus aureus* (MRSA) decolonization is an intervention aimed at reducing transmission of a specific MDRO although it is worth remembering that this intervention will also reduce methicillin-susceptible *S. aureus* colonization. There is still debate about the role of nasal mupirocin to reduce MRSA carriage, including who to screen and when, and how to manage recolonization, which is seen in up to 50% of neonates. A Society for Healthcare Epidemiology of America (SHEA) white paper in 2020 suggested that neonatal strategies for bacterial decolonization target specific contexts (e.g., outbreaks) or populations (e.g., patients at high risk of infection) (41). The evidence supporting nasal mupirocin is mixed, with some reports of successful reduction and elimination of MRSA colonization, infection, or both (42). Others argue that alternative approaches such as cleaning (43) or adoption of a multimodal approach, including strict cohorting, colonization screening, improved hand hygiene, and environmental cleaning compliance can reduce MRSA transmission (44). Whole-body CHG bathing is an example of an intervention aimed at reducing bacterial transmission and colonization in general (45–47), although the evidence for reducing HAI and MDRO infection is conflicting (48, 49).

##### Hand Hygiene

The hands of healthcare workers are frequently contaminated in the course of healthcare delivery and are often implicated in pathogen transmission (50, 51). Hand hygiene is identified as a key HAI prevention strategy by the WHO and most national IPC guidelines, with demonstrated success in improving hand hygiene compliance in resource-limited settings. However, there is limited evidence to improve hand hygiene compliance and prevent HAI in resource-limited neonatal units (52–55). As parents provide a large proportion of daily neonatal care and contribute a high number of hand hygiene opportunities, any hand hygiene intervention should involve these important caregivers (55).

##### Screening for MDROs

Multi-drug-resistant Gram-negative bacteria, such as *Klebsiella pneumoniae* and *Acinetobacter baumannii*, are the predominant HAI pathogens in LMIC neonatal units (56–59). In sub-Saharan Africa, neonatal rectal MDRO colonization rates have been shown to exceed 50% in some settings, with carriage rates rising rapidly the following hospitalization (47, 60, 61). Active patient screening for MDRO colonization at admission or at regular intervals during hospitalization may facilitate early identification, isolation, and application of contact precautions to reduce potential cross-infection within neonatal units (62). However, most resource-limited neonatal units have few or no isolation facilities, complicating the ability to separate MDRO-colonized neonates from non-colonized neonates. Given the widespread and hyperendemic nature of MDRO colonization in hospitalized LMIC neonates, active screening may be particularly useful during outbreaks or for limited surveillance approaches [e.g., monthly point prevalence surveys (PPS)].

##### The Hospital Environment: Built, Aqueous, and Equipment

Overcrowding is often cited as a contributing factor to pathogen transmission in neonatal units (63–65), but efforts to redesign hospital environments to reduce crowding and facilitate other IPC measures have not been rigorously studied in LMICs. In high-income countries, single family rooms/units (vs. cohort wards/cubicles) have lowered HAI rates (66, 67). However, single patient rooms are neither feasible nor safe in under-resourced neonatal units due to a lack of healthcare personnel and monitoring devices. Modular walls and movable barriers allow flexible cohorting by MDRO colonization or infection status, with evidence of a modest reduction in horizontal infection transmission (68, 69). In resource-limited neonatal units, incubators have been used successfully for patient “isolation” in the absence of formal isolation rooms or areas.

Sink drains, washbasins, and other moist surfaces (e.g., humidifiers and ventilator tubing) are sources of nosocomial outbreaks in neonatal units (70–73). Potential interventions to prevent colonization with waterborne pathogens include waterless hand hygiene using CHG or alcohol-based hand sanitizer (74), water-free patient care including sink removal from patient care areas (75, 76), and acetic acid (vinegar) as a low-cost method to decontaminate sink drains and multiple-use medical equipment (77–79).

Prevention of horizontal MDRO transmission in neonatal units relies on the implementation of robust IPC practices, including cleaning and disinfection of high-touch surfaces and equipment (80). While cleaning and disinfection can reduce the burden of pathogens in the neonatal environment (70), implementing and sustaining adequate environmental cleaning remains a challenge. High-touch surfaces may become contaminated between cleaning times, undermining hand hygiene initiatives by causing hand recontamination in the neonatal care unit. The use of low-cost tools, such as fluorescent markers or gel dots, to assess cleaning quality and provide regular feedback is relatively easy to implement in many LMIC settings. This strategy, within a multimodal bundle, has been demonstrated to improve cleaning practices (70, 81, 82). Commonly used neonatal medical and feeding equipment, such as suction catheters (83–85) and breast pumps (86, 87), are also prone to contamination, especially when shared or reused. Potential interventions include multimodal cleaning interventions (70), nurse training in disinfection methods, (79) and using surface materials with antimicrobial properties (88).

##### Skin Antisepsis

Interventions to improve skin integrity and antisepsis represent a crucial opportunity to prevent HAI and require greater clinical focus and further research. As the largest organ in the greatest contact with the external environment, neonatal skin is an important area of vulnerability, particularly in preterm neonates. However, evidence to guide the best interventions for skin antisepsis and integrity remains limited. Skin antisepsis prior to invasive procedures is well established, but there is a significant variation in the practice of the use of antiseptics for the prevention of neonatal sepsis. There is no consensus on the best antiseptic and concentration to use. Considerations must include balancing efficacy and safety concerns, particularly in extremely preterm neonates who are at risk of severe skin reactions (89–93).

Chlorhexidine gluconate is widely used for skin preparation prior to invasive procedures (94), but in addition to uncertainties about the optimal concentration, the question of whether to combine with alcohol (as recommended in older children/adults) is the subject of ongoing research (95). Other less commonly used antiseptics such as octenidine and povidone-iodine are also being studied, although the latter is associated with a risk of thyroid dysfunction (96). Importantly, increasing documentation of tolerance of Enterobacterales (*including Klebsiella* spp.) and *Staphylococcus* spp. to antiseptics, including CHG and octenidine, may decrease their effectiveness and necessitate the use of higher antiseptic concentrations (92). In addition, the efficacy of CHG and other antiseptics for the prevention of neonatal sepsis in most LMICs where Gram-negative pathogens predominate is unclear (47). Aside from antisepsis for invasive procedures, there is strong evidence for prophylactic CHG (4%) application to the umbilical cord in the community in high mortality settings (97), and for reduction of infection and mortality with CHG bathing (2%) within the multiple bundled IPC interventions in hospitalized neonates (98). There is a clear need for more evidence on whole-body prophylactic antiseptic use in high-risk hospitalized neonates, including optimal type, frequency, and concentration.

## Maintenance of Skin Integrity

The promotion of neonatal skin integrity requires multifaceted approaches that include rationalizing medical procedures, minimizing harm from devices and medical adhesives (99), positioning to prevent pressure sores, careful diapering, using barrier creams, providing skin-to-skin and KMC, as well as optimizing humidity, temperature, and nutrition (100, 101). Beyond this, generalized emollient application, which improves neonatal skin condition (102), is also receiving increased research focus using either synthetic or natural products. Emollient-impregnated wipes have been demonstrated to improve skin conditions, but may be prohibitively expensive (103). Importantly, some studies have suggested that the use of certain emollients may be associated with increased colonization by *Staphylococcus* spp. and increased rates of candidaemia (100). Alternatively, natural oils have been in longstanding traditional use, but some oils may be harmful (104, 105) while others have advantages with particular fatty acid profiles (e.g., higher linoleic acid content) (106–108). The use of sunflower oil as an emollient demonstrated a reduction in infections in neonates in a hospital-based study (109), and encouraging trends in a subgroup analysis of very low birth weight infants in a community-based study (106); however, more evidence is required (110). The strategy of combining emollients with antiseptics has also been the subject of recent pilot studies (47) and warrants further research.

## HAI Surveillance, IPC Education, and Advocacy

### Advocacy for Infection Prevention and Surveillance Programs in LMIC Neonatal Units

Low- and middle-income countries' neonatal units face many challenges, such as inadequate financing, understaffing, aging infrastructure, and the lack of effective leadership (8). IPC is the foundation of safe healthcare service provision for vulnerable populations. Prioritization of IPC programs is likely one of the most effective in-hospital public health interventions for LMICs striving to reduce neonatal mortality. Support and guidance on developing and sustaining neonatal unit IPC programs are needed from national and provincial ministries of health, institutional managers, neonatal nurses, and physician leaders alike. Medical and nursing societies are an often untapped area of support for clinicians and infection control practitioners. Benefits involve sharing expertise, networking beyond one's own place of work, developing guidelines, and advocacy. A recent example is a supplement on antimicrobial stewardship and IPC for LMICs (111).

### Enhanced HAI Surveillance and Feedback

Institution of infection surveillance in neonatal units is a priority for patient safety and quality of care (112, 113). Coordinated infection surveillance programs and regular IPC practice audits are key activities to assist with the identification of neonatal units with high HAI burden. Surveillance on its own can contribute to the reduction of HAIs (114) and can be an effective mechanism for early detection and control of HAIs including outbreaks (113, 115). HAI surveillance establishes infection rates and trends, detects outbreaks, and measures the risk of infection. Any HAI surveillance plan should yield accurate and comprehensive information within the limits of available resources (116). Surveillance data should ideally include stratification by infection timing (early onset sepsis vs. HAI), infection type, or site (bloodstream, urinary tract infection or meningitis; laboratory-confirmed vs. clinically suspected), and perinatal risks of infection (birth weight, gestation, comorbidities, anatomical anomalies, invasive procedures, and indwelling devices) and causative pathogens with their antimicrobial susceptibility profile (when available).

Although prospective HAI surveillance is the gold standard, it is neither feasible nor sustainable in many LMICs. Also, many low-resource settings lack diagnostic microbiology services, which are key to implementing prospective surveillance (32). An alternative method of surveillance, PPS using clinical definitions of HAI, although less robust, is less expensive and easier to conduct in resource-limited settings (117, 118). Ideally, PPS should focus on a limited spectrum of high-impact neonatal HAI (such as bloodstream infections and meningitis) and be coupled with ongoing improvement efforts. Also, PPS protocols using clinical definitions should be considered for settings that lack access to diagnostic microbiology services but may overestimate the HAI burden.

Regular audits of compliance with IPC standard operating procedures should also be conducted. The audit tool must be customized so that observations align with the unique practices and risks particular to neonatal care (119). Other performance data that provide valuable information on IPC risk are hand hygiene compliance audits, compliance with IPC interventions (e.g., care bundles), and adverse events in relation to IPC. All of these data sources must be evaluated in conjunction so that priorities for performance improvement can be determined and targeted interventions can be implemented. Regular feedback to staff about performance and HAI outcomes helps to reinforce IPC education, maintain staff engagement, create a culture of compliance, and modify behavior (120, 121).

### Staff and Parent Education

Healthcare staff training should be based on up-to-date IPC standard operating procedures for the healthcare institution, ensuring that all staff follows expected standards of care. To ensure compliance with IPC standards, regular in-service training accompanied by the assessment of knowledge and performance is essential. Teaching methods should be varied and aimed at achieving active participation, promoting insights, and eventually effecting behavior modification. IPC education is a key element in the success of multimodal HAI performance improvement initiatives (122). As mothers are constantly present in the neonatal unit, their importance in IPC should be recognized and incorporated into standards of care, IPC education, and monitoring programs. Topics should include hand hygiene, infection transmission between babies, the need for clean equipment and clean surfaces around the baby, maintaining good personal hygiene, handling of expressed breast milk and formula feeds, and reporting to medical staff if they become ill.

## Infection Prevention Bundles and Programs for LMIC Neonatal Units

Bundling care interventions to prevent neonatal sepsis in LMIC has great potential. Care bundles simplify and enable the reliable application of evidence-based best practices, typically applying four–six evidence-based interventions simultaneously (123). Ideally, bundle elements should be supported by data from randomized controlled trials, although evidence from peer-reviewed, indexed research papers is acceptable where trial data are lacking (124). To date, most bundles have targeted patients in intensive care settings and the prevention of device-associated infections (124). In neonates, bundles have been used successfully to prevent ventilator-associated pneumonia (VAP) and central line-associated bloodstream infections (CLABSIs) (125).

Adaptation of care bundle methodology, disease, and device targets is needed to ensure broad applicability in LMIC neonatal units. In these settings, programs to prevent device-associated infections (VAP and CLABSI) may be less important than bundles targeting more commonly used devices [e.g., peripheral intravenous cannulas (PIVCs)] and procedures (e.g., noninvasive ventilation). PIVC insertion is the most frequently performed procedure in neonatal units (126, 127). This procedure, which increases the risk of infection, commonly requires multiple insertion attempts before successful cannulation (126, 128, 129), and PIVCs are often replaced due to a short lifespan (129–131). Therefore, the need for a PIVC should be critically evaluated for each neonate and these devices should be removed as soon as feasible. Additionally, measures to prolong functional duration, such as effective dressing and securement, may be suitable targets for interventions.

**Figure 1:**
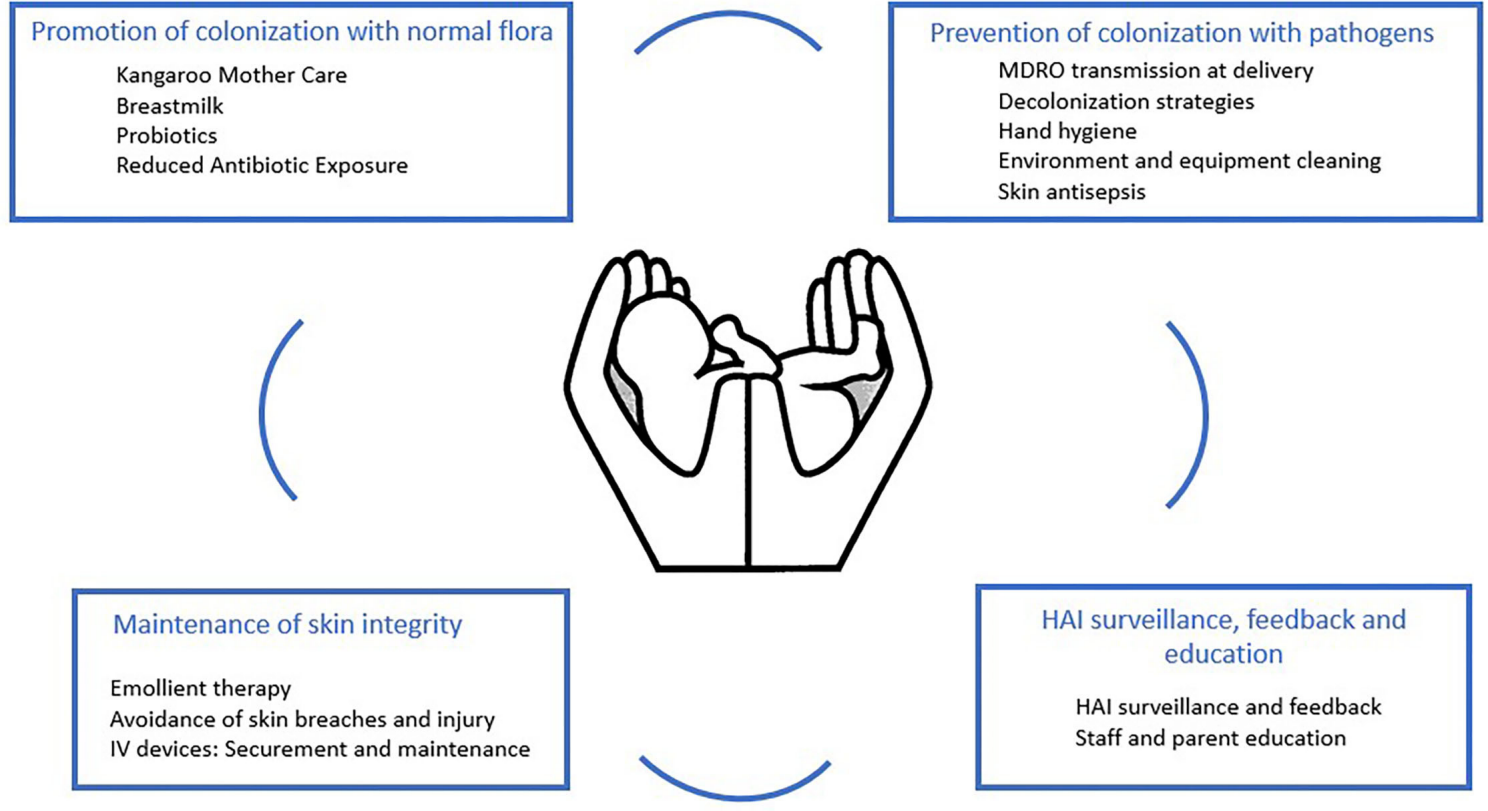
Key interventions for the prevention of healthcare-associated infections in neonates.

Individual components of neonatal HAI prevention care bundle in LMICs should be low-cost or highly cost-effective, simple to implement, measure and evaluate, and scalable. Further important considerations are the cultural acceptability of any intervention and the involvement of mothers who perform a substantial amount of in-hospital neonatal care in many resource-limited facilities. Lastly, any neonatal HAI prevention bundle should include interventions targeting multiple mechanisms of sepsis prevention (Figure 1). Possible bundle elements could include the promotion of a clean birthing and neonatal unit environment, CHG body washing, emollient application, PIVC access care, and interventions to improve hand hygiene compliance in neonatal caregivers, among others. The development of HAI prevention bundles and programs suitable for use in LMIC neonatal units should be prioritized as the evidence base supporting these interventions is currently extremely limited (Table 1).

A systematic review describing interventions to reduce hospital-acquired bloodstream infections in neonates in LMIC was recently published, including both single and bundled interventions (132). Overall, there was limited evidence, with no studies from low-income settings, only one from sub-Saharan Africa (98) and just two based in multiple countries (133, 134). Among the reports of studies using a single intervention, the strongest evidence was for sunflower oil emollient, which reduced neonatal bloodstream infections in Egypt and Bangladesh (108, 135). There was limited evidence for KMC in reducing bloodstream infection, of which only one demonstrated an impact on infection (136, 137), in contrast to a previous Cochrane review (13).

Of the nine studies that evaluated bundled interventions (of which seven are LMIC), the strongest evidence was for the prevention of device-associated infection, namely, VAP and CLABSIs. VAP rates were reduced in a multicountry study in 10 NICUs [relative risk (RR) 0.67; 95% confidence interval (CI) 0.50–0.91], after the implementation of a multimodal strategy including hand hygiene, oral antiseptics, ventilator circuit management, and enhanced infection surveillance (133), with similar reductions seen in smaller single-site studies in China and Egypt (138, 139) CLABSIs were also reduced in a four-country study using staff feedback, education, and surveillance (134), and in one single-site study using hand hygiene, CHG skin preparation, and education (140). Encouraging evidence from a single-site study in Zambia evaluated a bundle of training, hand hygiene promotion, text message reminders on IPC practices, enhanced cleaning, and weekly bathing of neonates >1.5 kg with 2% CHG (49, 98). Overall, the bundle significantly reduced neonatal mortality risk by 21% (RR 0.79; 95% CI 0.76–0.83), culture-proven, and clinically suspected bloodstream infection in all but the smallest babies (<1 kg).

## Conclusion

To address the high morbidity and mortality of neonatal HAI in LMIC, IPC programs and evidence-based specific HAI prevention interventions should be identified and prioritized by institutions and national ministries of health. For the greatest impact, these interventions should be implemented simultaneously in care bundles or multimodal programs and target multiple mechanisms of sepsis prevention.

## Author Contributions

AD drafted the manuscript outline, solicited contributions from each co-author, and compiled the edited first draft of this manuscript. All authors discussed and planned the manuscript content, provided substantive feedback on the first draft and coedited this manuscript, and approved the final version and agree to be accountable for the content of this work.

## Funding

AD is supported by a National Institutes of Health Emerging Global Leader Award (NIH K43 TW010682). FF is supported by the Academy of Medical Sciences and by funders of the Starter Grants for Clinical Lecturers scheme and the National Institute for Health Research Great Ormond Street Hospital Biomedical Research Centre.

## Conflict of Interest

The authors declare that the research was conducted in the absence of any commercial or financial relationships that could be construed as a potential conflict of interest.

## Publisher's Note

All claims expressed in this article are solely those of the authors and do not necessarily represent those of their affiliated organizations, or those of the publisher, the editors and the reviewers. Any product that may be evaluated in this article, or claim that may be made by its manufacturer, is not guaranteed or endorsed by the publisher.
